# Effects of CO_2_ Curing on Alkali-Activated Slag Paste Cured in Different Curing Conditions

**DOI:** 10.3390/ma12213513

**Published:** 2019-10-26

**Authors:** Yubin Jun, Seong Ho Han, Tae Yong Shin, Jae Hong Kim

**Affiliations:** Department of Civil and Environmental Engineering, Korea Advanced Institute of Science and Technology, Daejeon 34141, Korea; ssjun97@gmail.com (Y.J.); ha8890@kaist.ac.kr (S.H.H.); tyshin@kaist.ac.kr (T.Y.S.)

**Keywords:** alkali-activated slag, CO_2_ curing, carbonation curing, calcite, vaterite, C-S-H

## Abstract

The effect of CO_2_ curing on alkali-activated slag paste activated by a mixture of sodium hydroxide and sodium silicate solutions is reported in this paper. The paste samples after demolding were cured in three different curing environments as follows: (1) environmental chamber maintained at 85% relative humidity (RH) and 25 °C; (2) 3-bar CO_2_ pressure vessel; and (3) CO_2_ chamber maintained at 20% CO_2_ concentration, 70% RH and 25 °C. The hardened samples were then subjected to compressive strength measurement, X-ray diffraction analysis, and thermogravimetry. All curing conditions used in this study were beneficial for the strength development of the alkali-activated slag paste samples. Among the curing environments, the 20% CO_2_ chamber was the most effective on compressive strength development; this is attributed to the simultaneous supply of moisture and CO_2_ within the chamber. The results of X-ray diffraction and thermogravimetry show that the alkali-activated slag cured in the 20% CO_2_ chamber received a higher amount of calcium silicate hydrate (C-S-H), while calcite formed at an early age was consumed with time. C-S-H was formed by associating the calcite generated by CO_2_ curing with the silica gel dissolved from alkali-activated slag.

## 1. Introduction

Greenhouse gas emission, including CO_2_, is one of the causes of global warming, which increases the frequency and extent of natural disaster in the world. Cement clinker production for Portland cement contributes 4% of global CO_2_ emission according to the statistics of 2017 [1], which is taken seriously in the construction industry. As environmental problems of CO_2_ emission becomes serious worldwide, there have been considerable efforts to find a way to reduce the CO_2_ level in the atmosphere. The construction industry has also tried to reduce greenhouse gas emissions.

The use of CO_2_ for accelerated curing of cement-based materials is one of the active responses to decrease CO_2_ concentration in the atmosphere. Calcium silicates such as alite and belite in Portland cement are spontaneously carbonated, which mainly results in the formation of calcium carbonate (CaCO_3_). Cement-based materials subjected to CO_2_ curing at an early age show a rapid development of their strength because in the process of CO_2_ curing, CaCO_3_ precipitates in pores of cast mortar and concrete. Densifying the pore refines their microstructure, including the cement paste matrix and the interfacial transition zone, which results in a higher strength at a rather early age [2,3]. However, previous studies adopted a very low water-to-cement ratio (w/cm) ranging from 0.06 to 0.28 [4], 0.11 to 0.25 [2], and 0.125 [5] to 0.18 [3]. This limits the application of CO_2_ curing in practice [3].

On the other hand, the use of alternative cement replacing Portland cement expectedly contributes to the decrease in CO_2_ emission. Calcium sulfoaluminate cement has merit due to the low temperature requirement for its calcination process; an approximately 100–200 °C decrease in the calcination temperature compared to the calcination for Portland cement contributes to lower CO_2_ emissions [6,7]. In addition, alkali-activated binders, including geopolymers synthesized with industrial byproducts such as blast-furnace slag and fly ash, are one of the promising alternatives [8,9,10,11]. It had been reported that they possesses excellent durability [11]. The blast-furnace slag utilized as a raw material also contains a large amount of calcium silicates. It is susceptible to carbonation while its hydration is latent, requiring an alkaline activator. The alkaline activator enables calcium to dissolve from slag particles, and the calcium participates in the formation of calcium silicate hydrate (C-S-H) gels contributing to the development of strength [12,13]. In addition, it is expected that the dissolved calcium could participate in the formation of CaCO_3_ in the process of CO_2_ curing. 

From the viewpoint of recycling industrial byproducts as well as a reduction in CO_2_ emission, this paper conducted research on CO_2_ curing for alkali-activated slag. The setting of a slag paste was obtained by the alkali activation, and the cast samples were subjected to CO_2_ curing. The CO_2_ curing was carried out in a pressure vessel of approximately 300 kPa of CO_2_ pressure or in a chamber maintained at a constant 20% CO_2_ concentration. The compressive strengths of the samples were studied according to the CO_2_ curing condition. X-ray diffraction (XRD) and thermogravimetric (TG) analyses on the samples quantitatively analyzed the quantity and crystallization of the reaction products.

## 2. Experiments

### 2.1. Materials

**Figure 1:**
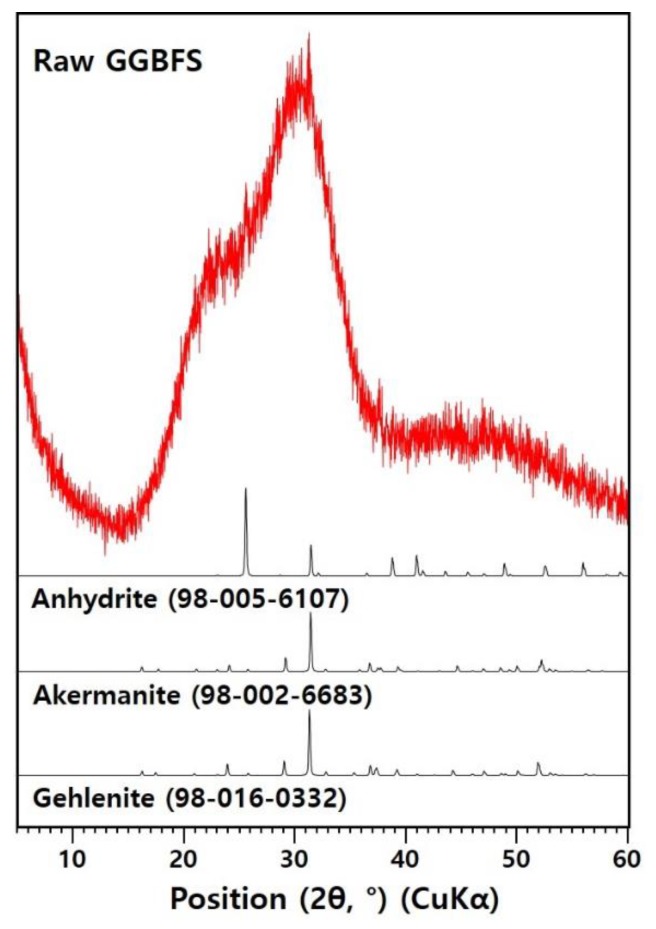
X-ray diffraction (XRD) patterns of raw GGBFS.

Ground-granulated blast-furnace slag (GGBFS) used in this study was produced by H Steel Company in Dangjin, Korea. Table 1 reports the chemical composition of GGBFS by X-ray fluorescence analysis. The GGBFS consists of lime and silica to a high extent. Figure 1 presents the XRD pattern of the GGBFS, which corresponds to the general pattern of blast-furnace slag [14,15,16]. Sodium hydroxide and sodium silicate were used as an alkaline activator. Sodium hydroxide pellets (NaOH; reagent grade higher than 98% purity) and liquid sodium silicate (Na_2_SiO_3_; reagent grade with a molar ratio of SiO_2_/Na_2_O of 2.8) were acquired from S company in Pyeongtaek, Korea.

### 2.2. Experimental Design and Sample Preparations

The mixture proportions of samples are shown in Table 2. In this study, alkali activators (i.e., activator type and activator concentration) were chosen to harden samples within 1 h for the given GGBFS. The alkaline activator was prepared by blending 5 M NaOH solution with liquid Na_2_SiO_3_. The blend ratio of the NaOH solution to the liquid Na_2_SiO_3_ was 1.0 by mass. The 5 M NaOH solution was prepared by dissolving NaOH pellets in deionized water. The weight ratio of the alkali activator (5M NaOH + Na_2_SiO_3_) to the binder (GGBFS) was 0.4.

After 4 min of mixing with a planetary mixer, the fresh paste was cast in 25 mm cube molds. Each sample was taken from a mold after 1 h of pre-curing in an environmental chamber at 85% relative humidity (RH) and 25 °C. Table 3 summarizes the designed curing conditions including the control of conventional humid curing (85% RH and 25 °C). The first set of samples (denoted with CO2P-##) was subjected to 99.9% purified CO_2_ in a pressure vessel, where the initial pressure was set between 3 and 4 bar. The demolded samples were placed in the pressure vessel and then the pressure vessel was vacuumed before injecting the CO_2_. The 3 bar CO_2_ curing continued for 3 h, 23 h or 167 h, and additional CO_2_ was injected in the middle of the 167 h curing. Pressure loss in the pressure vessel was monitored using a pressure digital gauge (PDR1000; Pressure Development of Korea Co., Daejeon, Korea). The sampling rate for the pressure measurement was 1 record per second. A total curing time of 4 h, 24 h, and 7 days (168 h) included 1 h of pre-curing. After the 3 bar CO_2_ curing, the samples were placed in the environmental chamber (85% RH and 25 °C) for further hydration. The samples cured for 3 h, 23 h, and 167 h in the 3 bar CO_2_ pressure vessel were labelled CO2P-T1, CO2P-T2, and CO2P-T3, respectively. The other sample, labeled CO2-HC, was cured in a CO_2_ chamber controlled at 20% CO_2_ concentration, 70% RH and 25 °C until its strength measurement.

The compressive strengths of the samples were measured at the age of 4 h, 24 h, 7 days, and 42 days after the mixing (Table 3), where an average of the strengths of four replicated specimens was reported. The strength development of alkali-activated slag paste was faster than normal cement concrete. Approximately 90% of the strength at 42 days was obtained even with a 7-day old sample. (see Table 4). This study therefore conducted a detailed investigation until 7 days for CO_2_ curing. After each measurement of compressive strength at the age of 4 h, 24 h, and 7 days, fractured specimens were finely powdered and then immersed in isopropanol to stop their hydration. The XRD and TG analyses were applied to the powdered samples after vacuum drying. The XRD and TG were performed for the phase analyses of the reaction products formed in samples. The XRD measurement was carried out using a high-power X-ray diffractometer (Rigaku Corp., Tokyo, Japan) with an incident beam of Cu Kα radiation for a 2θ scanning range of 5–60°. The XRD patterns of samples were analyzed with the International Center for Diffraction Data (ICDD) PDF-2 database [17] and the Inorganic Crystal Structure Database (ICSD) [18]. The TG measurement was performed in nitrogen gas at a heating rate of 10 °C/min from room temperature to 950 °C using an SDT Q600 thermal analyzer (TA Instruments, Inc., New Castle, Delaware, USA).

## 3. Results and Discussion

**Figure 2:**
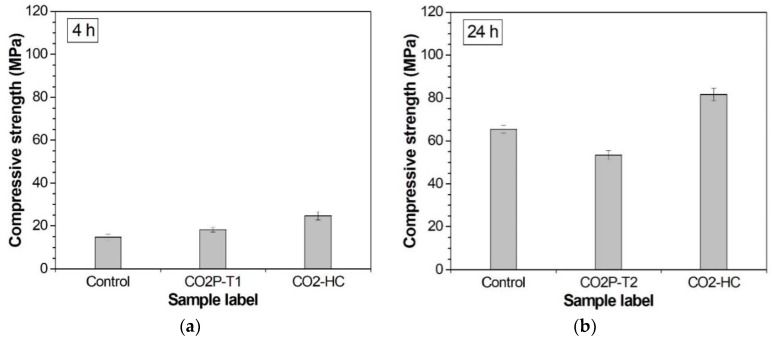
Compressive strengths at 4 h (**a**) and 24 h (**b**).

CO_2_ curing reportedly accelerates the strength development of Portland cement-based materials [5,19]. Alkali-activated slag pastes were cured in various CO_2_ curing environments, and Table 4 reports the effect on their compressive strength. Figure 2 directly compares the effect of CO_2_ curing at the moment when curing finished. The time shown in Figure 2 includes the 1 h pre-curing required to have acceptable demolding in common (Table 3). The effect of CO_2_ curing for 3 h was compared in Figure 2a: the sample cured in the 20% CO_2_ concentration chamber (CO2-HC) showed the highest strength, followed by the sample cured in the 3 bar CO_2_ pressure vessel (CO2P-T1). Both CO_2_ curing conditions provide a higher strength than the control curing environment. However, a longer period of CO_2_ curing had a negative effect on strength. As seen in Figure 2b, the samples cured in the 3 bar CO_2_ pressure vessel (CO2P-T2) for 23 h gave a lower compressive strength than the control sample. In contrast, the samples cured in the 20% CO_2_ concentration chamber still showed a higher strength than the control. The trend on the strengths at 168 h (CO2P-T3 for 167 h) was the same as CO2P-T2 for 23 h, as shown in Table 4.

**Figure 3:**
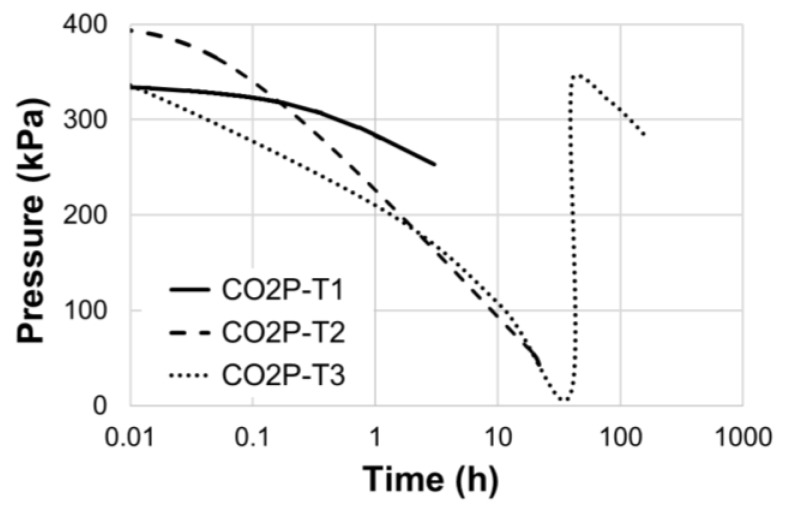
Pressure loss in the pressure vessel during CO_2_ curing.

Here it should be noted that CO_2_ was sufficiently supplied for CO_2_ curing. Figure 3 shows the measured pressure loss in the pressure vessel during CO_2_ curing. The CO_2_ pressure decreased over time while the initial pressure was controlled between 3 and 4 bar as previously described. This implies that the CO_2_ was consumed with time. About 24 h after injecting CO_2_, the CO_2_ pressure dropped to a level of approximately 0.3 bar. In this study, CO_2_ was reinjected so that the CO_2_ pressure did not drop to 0 bar during CO_2_ curing. For the CO2P-T3 case (CO_2_ curing for 167 h), CO_2_ was reinjected one time during the 167 h of CO_2_ curing (Figure 3).

**Figure 4:**
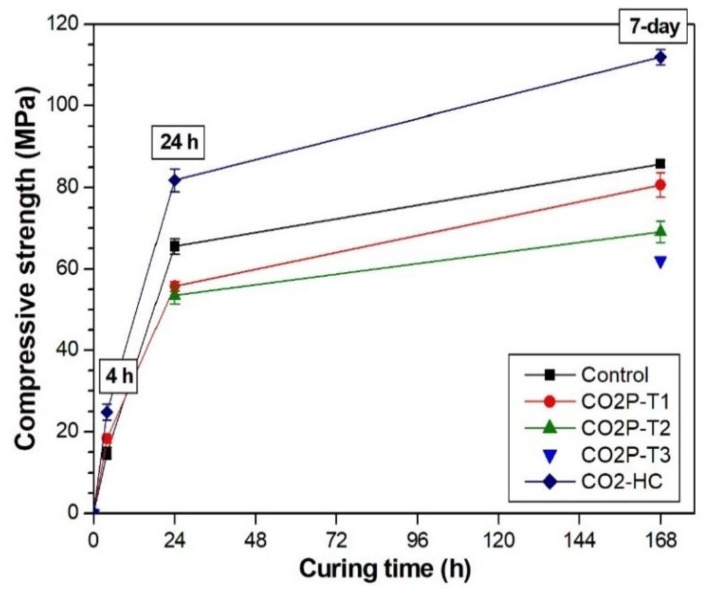
Compressive strength developments of paste samples cured under different curing conditions with curing time.

A successive moisture curing after CO_2_ curing reportedly causes the hydration of calcium silicates, which results in further increases in the strength of Portland cement-based materials [2,20,21]. Figure 4 presents the compressive strength development of the samples taken after further moisture curing following CO_2_ curing. In the case of the CO2-HC samples, since they were cured in a 20% CO_2_ concentration chamber maintained at 70% RH, we can presume that the samples were subjected to moisture curing. It achieved the highest strength of 111.89 MPa at seven days while the control alkali-activated slag reached 85.77 MPa at seven days. Further hydration on the samples cured in the 3 bar CO_2_ pressure vessel (CO2P-T1) obviously provided strength development, but its increase was lower than the control. The strength of the control sample exceeded that of CO2P-T1 at 24 h and seven days while the 4 h strength of the control sample was lower than that right after CO_2_ curing. 

It was again confirmed that the long period of CO_2_ curing (CO2P-T2 for 23 h and CO2P-T3 for 167 h) in the 3 bar CO_2_ pressure vessel was not effective. Their compressive strengths were lower than that of the control sample. The effect of further moisture curing on the CO2P-T2 sample was also not effective.

**Figure 5:**
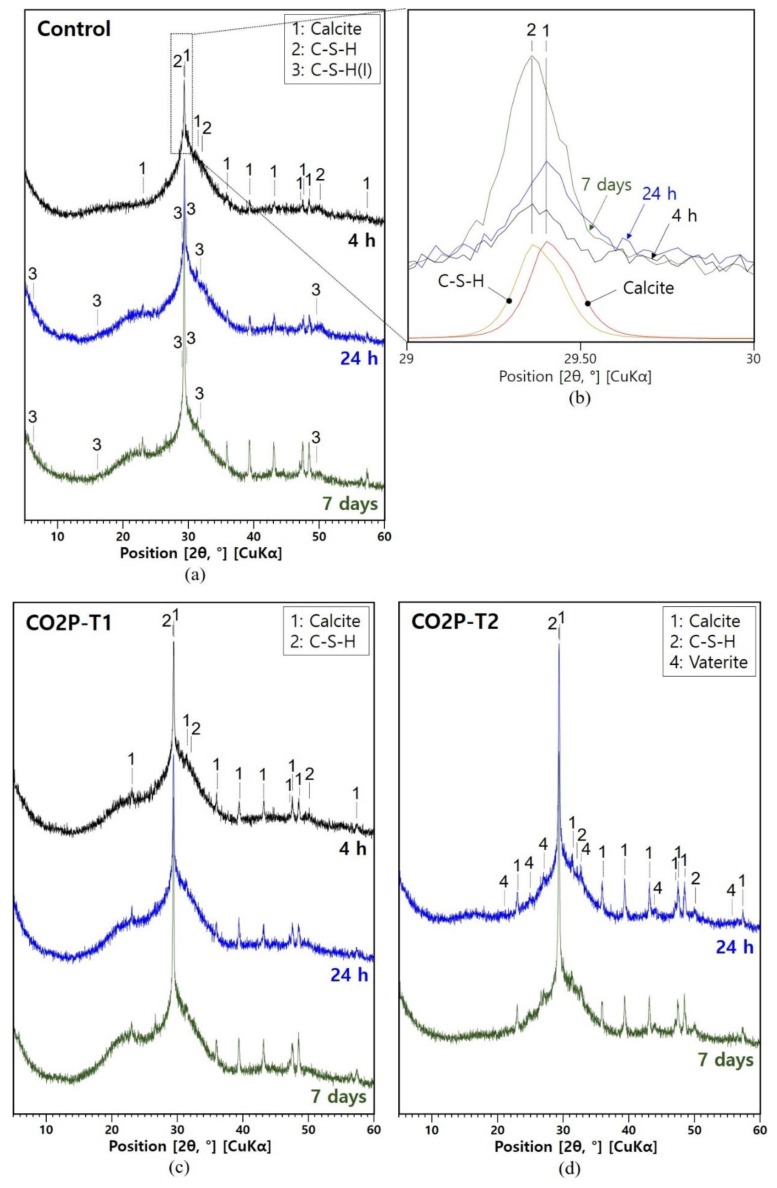

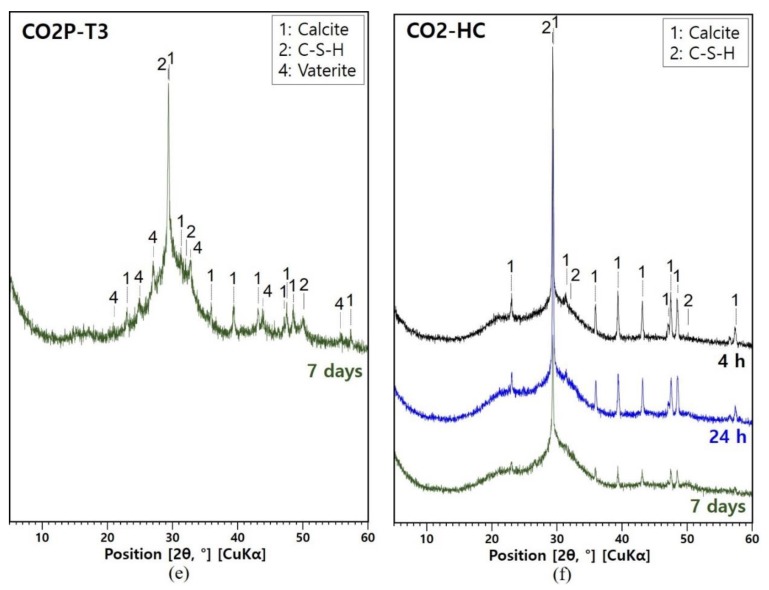
XRD patterns of samples with curing time. (**a**) Control; (**b**) detailed XRD figure of the control sample in the 29–30° 2θ range with reference patterns of calcite and calcium silicate hydrate (C-S-H); (**c**) CO2P-T1; (**d**) CO2P-T2; (**e**) CO2P-T3; (**f**) CO2-HC. 1: calcite (PDF 98-005-2151), 2: C-S-H (PDF 00-033-0306), 3: C-S-H(I) (PDF 00-029-0331), and 4: vaterite (PDF 98-018-1959).

The XRD patterns of all samples are shown in Figure 5. Earlier studies have shown that calcite, C-S-H, hydrotalcite, and calcium alumina silicate hydrate (C-A-S-H) were present in NaOH/Na_2_SiO_3_-activated slag [12,22,23]. It was also reported that the reaction products of alkali-activated slag depend on the composition of slag and the activator. Nevertheless, C-S-H is the main reaction product in alkali-activated slag [12,13] regardless of the activator used. As shown in Figure 5a, the main reaction products of the control sample were calcite and C-S-H. Figure 5b shows the detailed XRD pattern, where the strongest peaks of calcite (29.395° 2θ) [18] and C-S-H (29.356° 2θ) [17] were overlapped at 29° to 30° 2θ. The formation of C-S-H(I), which is a more crystalline form of C-S-H [24], was additionally found at 24 h and seven days.

CO_2_ curing for the alkali-activated slag pastes (Figure 5c–f) also provided calcite and C-S-H as their main products. However, C-S-H(I) was not present in the CO_2_-cured samples until seven days. In addition, vaterite, a metastable allotropic form of CaCO_3_ [25,26,27], was found in the samples right after the 3 bar CO_2_ curing (CO2P-T2 at 24 h and CO2P-T3 at seven days). The vaterite was not found in CO2P-T1 (the 3 bar CO_2_ curing for 3 h) and CO2-HC (the continuous CO_2_ curing in the 20% CO_2_ chamber).

**Figure 6:**
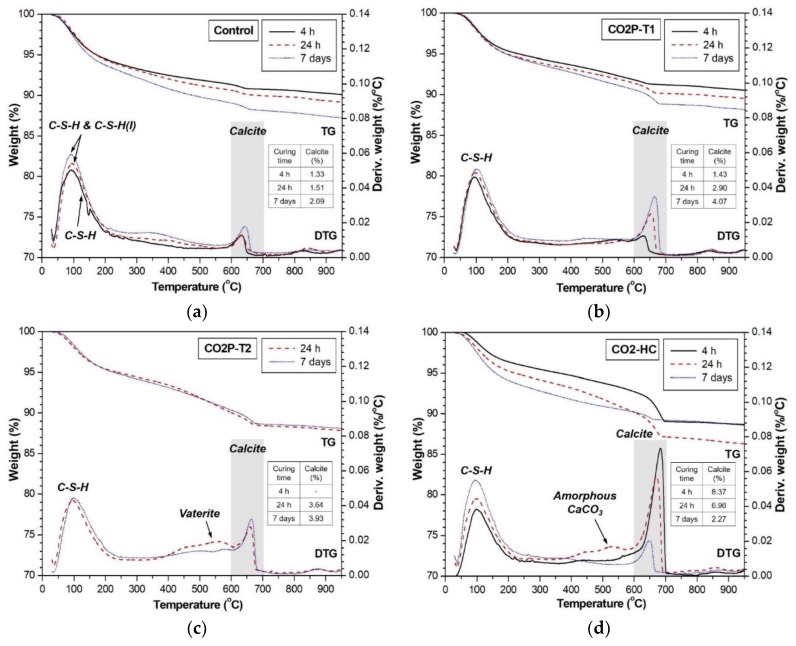
TG and derivative TG (DTG) curves of (**a**) control, (**b**) CO2P-T1, (**c**) CO2P-T2, and (**d**) CO2-HC samples with curing time.

TG and derivative TG (DTG) curves of the alkali-activated slag pastes before and after CO_2_ curing are compared in Figure 6. The first weight loss below 200 °C, clearly identified in the DTG curves, represented the loss of the combined water due to dehydration of C-S-H (50–200 °C [28]) and C-S-H(I) (90–110 °C [13]). The weight loss in the range of 600–700 °C indicated the presence of calcite [29,30]. The amount of calcite in each sample could be roughly quantified on the basis of the weight loss from 600–700 °C [29]. The calcite concentrations at all ages of the samples are tabulated in Table 5.

The extent of C-S-H in the control sample obviously increased with conventional humidity curing (see Figure 6a). Although the weight loss of C-S-H and C-S-H(I) overlaps, it can be shown that C-S-H increased with curing time because the C-S-H(I) peak in the XRD result was almost the same between 24 h and 7 days. This observation can also be identified in Figure 5b. In addition, the DTG peak of calcite slightly increased with curing time. The calcite concentrations were evaluated at 1.33%, 1.51%, and 2.09% at 4 h, 24 h and 7 day, respectively. It was reported that C-S-H in alkali-activated slag paste is also carbonated even in conventional humidity curing [31]. As a result, the formation of C-S-H and C-S-H(I) contributes to the strength of alkali-activated binders [29,32], and the formed calcite is helpful in improving the early age strength even though the concentration is as low as 2.8–4.6% [29]. The strength development of the control sample, shown in Figure 4, also supports the correlation. Furthermore, C-S-H is considered as the major reaction product contributing to strength development because the peak intensity of C-S-H(I) in the XRD pattern was relatively small and the growth rate of calcite was low, as reported in Table 5.

Even when the samples were cured in the 3 bar CO_2_ pressure vessel, we still observed a small amount of calcite. The calcite concentrations right after CO_2_ curing were only 1.43% for 3 h, 3.64% for 23 h and 2.76% for 167 h (CO2P-T1, CO2P-T2 and CO2P-T3, respectively) as reported in Table 5. CO_2_ in the gaseous phase does not react, and its dissolution in pore water is required for the formation of CaCO_3_ [31,33]. Water starvation due to dry-out of the samples was reported to decrease the carbonation [5]. In the case of alkali-activated materials, the high pH of the alkali activator can hinder the CO_2_ dissolution and obtain a low degree of carbonation.

In regards to porosity, carbonation of the inside of a cast sample needs CO_2_ diffusion into the sample, which can be obtained with a higher extent of air pores. Therefore, a low w/cm was adopted and then the produced mixes were compacted in the previous study. For example, w/cm ranges from 0.06 to 0.28 [4], 0.11 to 0.25 [2], 0.125 [5] or 0.18 [3]. Pre-conditioning for effective CO_2_ curing sometimes included an additional process to evaporate free water in the compacted samples [34,35]. As a result, the low w/cm compact sample with a high efficiency on CO_2_ curing had a high air-filled porosity beyond 20%. The samples produced in this study had a high activator (liquid)-to-binder ratio, and they had a low volume of air pores compared to the compact samples. Among them, the CO2P-T3 sample was cured for a sufficiently long time (167 h) that CO_2_ diffusion was expected inside. Nevertheless, it gave a low calcite concentration (2.76%). Therefore, the CO_2_ diffusion related to air-filled porosity cannot be considered as a critical factor.

Successive hydration of the sample after CO_2_ curing contributed to a higher amount of calcite as shown in Figure 6b. The calcite concentrations in CO2P-T1 increased over time: 1.43%, 2.90%, and 4.07% as reported in Table 5. The sample had a relatively higher degree of carbonation than the control sample even though the calcite concentration was still low (less than 5%). However, as shown in Figure 4, the strength of the CO2P-T1 sample was less than that of the control sample at all ages. The small amount of calcite formation by CO_2_ curing was negative on the strength of alkali-activated slag.

The 20% CO_2_ concentration curing provided a different trend of calcite formulation as shown in Figure 6d and Table 5. First of all, calcite formation in the short period (3 h) of CO_2_ curing was substantial: 8.37% at 4 h. Compared with the other samples, the high level of calcite concentration is attributed to the simultaneous presence of moisture and CO_2_ in the chamber. The RH was also controlled at 70%. The CO_2_ dissolution is more active with the neutral moisture supplied in the CO_2_ chamber. The highest concentration of calcite was confirmed at 4 h and then it decreased with curing time. It is worth noting that C-S-H formation kept increasing with curing time. During the hydration of alkali-activated slag paste, Ca as well as Si dissolved first in an alkaline environment and then C-S-H, including the other products that entered a solid phase in the paste. Ca^2+^ dissolved from the blast-furnace slag is preferably consumed for the calcite formation at an early age. The calcite formation is suppressed when the Ca dissolution in an alkaline environment exceeds the CO_2_ dissolution in the limited amount of moisture. The calcite is then consumed to form C-S-H with silica gel released from the blast-furnace slag: SiO_2_∙xH_2_O + yCaCO_3_ + H_2_O ↔ yCaO∙SiO_2_∙xH_2_O + H_2_CO_3_, as reported in [31]. The major reaction product of C-S-H was dominated in the CO2-HC sample, which results in the highest strength among the samples considered in this study.

On the other hand, in the case of the CO2P-T2 and CO2P-T3 samples, weight loss in the range of 520–580 °C was identified and it was related to the decomposition of vaterite [27]. The difference in the strength development with time in the sample (Figure 4) seems to be due to the formation of vaterite (metastable CaCO_3_). Among the samples cured under CO_2_ pressure, the samples that were cured for 23 h or 167 h showed the presence of vaterite, except for the sample that was CO_2_-cured for 4 h. This probably indicates that the CO_2_ curing time affects the polymorphs of CaCO_3_. As shown in Figure 6c, further humidity curing decreased the DTG peak of vaterite at seven days and increased the peak of calcite. Vaterite can be easily recrystallized to calcite when exposed to water [27].

The CO2-HC sample at 24 h also showed the weight loss in the range of 450–550 °C. This weight loss is likely due to the decomposition of amorphous calcium carbonate (CaCO_3_∙xH_2_O [36]) rather than vaterite, which is because the peaks for vaterite was not detected in the XRD result as shown in Figure 5f. Amorphous CaCO_3_ was reportedly decomposed at temperatures between 245 °C and 645 °C [37].

## 4. Conclusions

CO_2_ curing for alkali-activated slag paste is promising in the view of reducing CO_2_ emission in the construction industry. In this study, blast-furnace slag is activated with 5 M NaOH solution and liquid Na_2_SiO_3_. The alkali-activated slag paste cured in a 20% CO_2_ concentration chamber (70% RH, and 25 °C) shows a higher compressive strength than the control samples cured at 85% RH and 25 °C. A higher amount of calcite was confirmed in the CO_2_ cured samples via XRD and TG analyses. The simultaneous supply of water vapor and CO_2_ in the chamber contributes to the CO_2_ dissolution, which results in the initial formation of substantial calcite (at 4 h). Continuous CO_2_ curing allows us to generate more C-S-H by the hydration of the calcite and silica gel dissolved from blast-furnace slag. As a result, the CO_2_-cured samples show a decrease in calcite concentration while the amount of C-S-H increases with curing time. However, the strengths of alkali-activated slag cured in a 3 bar CO_2_ pressure vessel were lower than the control samples. CO_2_ is hardly dissolved at the high pH of the pore solution of the alkali-activated slag, and a lower amount of calcite is formed even after CO_2_ curing in the pressure vessel. Limiting the supply of moisture in the pressure vessel prohibits the hydration of alkali-activated slag as well as CO_2_ dissolution. The strength development of the alkali-activated slag cured in the CO_2_ pressure vessel is therefore lower than the control samples cured in a conventional humid environment. This study finally concludes that CO_2_ curing at a constant CO_2_ concentration was more effective on alkali-activated slag paste than in a 3 bar-CO_2_ pressure vessel.

## Figures and Tables

**Table 1:** Chemical composition (oxides in wt.%) of ground-granulated blast-furnace slag (GGBFS).

CaO	SiO_2_	Al_2_O_3_	Na_2_O	K_2_O	MgO	MnO	TiO_2_	SO_3_	P_2_O_5_	Fe_2_O_3_	Others
47.97	30.76	13.26	0.23	0.54	3.06	0.52	0.87	1.81	0.01	0.61	0.25

Note: Others include BaO, ZrO_2_, V_2_O_5_, SrO, and Y_2_O_3_.

**Table 2:** Mix proportions of samples.

Binder (g)	Activator (g)		Activator/Binder
GGBFS	5M NaOH	Liquid Na_2_SiO_3_	
2400	480	480	0.4

**Table 3:** Curing conditions of samples.

Sample Label	Curing time				
	1 h	3 h	20 h	6 Days	35 Days
Control	Chamber at 25 °C and 85% RH	Chamber at 25 °C and 85% RH			
CO2P-T1		3-bar CO_2_ pressure vessel	Chamber at 25 °C and 85% RH		
CO2P-T2		3-bar CO_2_ pressure vessel		Chamber at 25 °C and 85% RH	
CO2P-T3		3-bar CO_2_ pressure vessel			Chamber at 25 °C and 85% RH
CO2-HC		20%-concentration CO_2_ chamber at 25 °C and 70% RH			

Note. Demolding after 1 h pre-curing; Strength, XRD, and TG tests at 4 h: Control, CO2P-T1, and CO2-HC samples; Strength, XRD, and TG tests at 24 h: Control, CO2P-T1, CO2P-T2, and CO2-HC samples; Strength, XRD, and TG tests at 7 days: Control, CO2P-T1, CO2P-T2, CO2P-T3, and CO2-HC samples; Strength test at 42 days: Control, CO2P-T1, CO2P-T2, CO2P-T3, and CO2-HC samples.

**Table 4:** Compressive strengths of the paste samples.

Sample Label	Compressive Strength (Standard Deviation), MPa			
	4 h	24 h	7 Days	42 Days
Control	14.78 (1.33)	65.56 (1.88)	85.77 (0.88)	108.83 (3.52)
CO2P-T1	18.27 (1.1)	55.72 (1.14)	80.62 (2.99)	85.6 (1.62)
CO2P-T2	-	53.47 (2.09)	69.11 (2.6)	74.69 (1.9)
CO2P-T3	-	-	61.96 (0.48)	73.87 (2.57)
CO2-HC	24.81 (1.96)	81.77 (2.86)	111.89 (1.88)	121.98 (1.43)

**Table 5:** Calcite concentrations of the samples.

Sample Label	Calcite Concentration		
	4 h	24 h	7 Days
Control	1.33%	1.51%	2.09%
CO2P-T1	1.43%	2.90%	4.07%
CO2P-T2	-	3.64%	3.93%
CO2P-T3	-	-	2.76%
CO2-HC	8.37%	6.96%	2.27%
